# A new framework for growth curve fitting based on the von Bertalanffy Growth Function

**DOI:** 10.1038/s41598-020-64839-y

**Published:** 2020-05-14

**Authors:** Laura Lee, David Atkinson, Andrew G. Hirst, Stephen J. Cornell

**Affiliations:** 1 Department of Evolution, Ecology and Behaviour, University of Liverpool, Liverpool, UK; 2 School of Environmental Sciences, University of Liverpool, Liverpool, UK; 3 Centre for Ocean Life, National Institute for Aquatic Resources, Technical University of Denmark, Kemitorvet, 2800 Kgs, Lyngby, Denmark

**Keywords:** Ecology, Evolution, Computational models, Statistical methods

## Abstract

All organisms grow. Numerous growth functions have been applied to a wide taxonomic range of organisms, yet some of these models have poor fits to empirical data and lack of flexibility in capturing variation in growth rate. We propose a new VBGF framework that broadens the applicability and increases flexibility of fitting growth curves. This framework offers a curve-fitting procedure for five parameterisations of the VBGF: these allow for different body-size scaling exponents for anabolism (biosynthesis potential), besides the commonly assumed 2/3 power scaling, and allow for supra-exponential growth, which is at times observed. This procedure is applied to twelve species of diverse aquatic invertebrates, including both pelagic and benthic organisms. We reveal widespread variation in the body-size scaling of biosynthesis potential and consequently growth rate, ranging from isomorphic to supra-exponential growth. This curve-fitting methodology offers improved growth predictions and applies the VBGF to a wider range of taxa that exhibit variation in the scaling of biosynthesis potential. Applying this framework results in reliable growth predictions that are important for assessing individual growth, population production and ecosystem functioning, including in the assessment of sustainability of fisheries and aquaculture.

## Introduction

Body size is a fundamental characteristic of all organisms. Body size has received much attention from biologists owing to its widespread covariation with a plethora of ecological and evolutionary functions and physiological traits^1–9^. Understanding growth (i.e. the changes in body size over time) is fundamental to many areas of biology, as well as being crucial for industries based on animal and plant production. Accurate growth predictions are fundamental to aquaculture and production industries, for example, over- or underestimating species growth will result in unreliable predictions of production and hence revenue and profit for producers^10^. For example, modelling the growth rates of farmed tiger prawns, *Penaeus monodon*, under varying environmental conditions including temperature and pond age, allows for predictions of production rates, and hence profitability, in new farming locations^11^. Moreover, gaining knowledge of growth parameters can help to inform management plans, which are required for effective conservation management of target species in aquaculture or reducing pressure on natural populations^12^. For example, growth models have predicted parameter values associated with slow growth and long lifespan in *Stichopus vastus* which has helped inform restrictions on catch quotas to allow natural populations to recover^13^. In addition, understanding growth dynamics has been shown to be important for bivalve species in aquaculture and their use in mitigating eutrophication in coastal areas, for example, gaining accurate growth predictions of soft tissue can help the efficiency of mussel production that is required for eutrophic coastal waters^14^.

Methods for fitting growth curves to empirical data are applied extensively^15–25^, but many of these approaches can be taxon-specific and lack flexibility to capture variation in growth over ontogeny or between conditions^26^. We propose a new framework for fitting growth curves which applies a set of re-parameterisations of the von Bertalanffy Growth Function (VBGF). This framework improves on existing methods by allowing for growth-curve fitting to a wide range of taxa which may exhibit variation in rates of growth, including exponential and supra-exponential growers.

The VBGF has been used extensively to model growth for numerous taxa such as fish^27^, mammals^28^, birds^29^, invertebrates^30,31^ and dinosaurs^32^. It is a special case of the Richards model^19^ and is based on biological principles originally developed by Pütter^33^. The mechanistic interpretation of the VBGF has varied over time, but most commonly growth is argued to occur if the building up of materials prevails over the breakdown of materials^34,35^ as denoted by the differential equation:

1 $$\begin{array}{c}\frac{dm}{dt}=H{m}^{A}-K{m}^{B},\end{array}$$

where $m$ denotes mass, $t$ is time from birth, $A$, $B$ are the mass-scaling exponents of anabolism (synthesis of component materials) and catabolism (breakdown of component materials) respectively, and $H$ and $k$ are the coefficients of anabolism and catabolism, respectively^35^. The $H{m}^{A}$ term in Eq. (1) can represent the resource availability for growth in an organism, with the mass-scaling exponent $A$ often assumed to relate to the body-mass scaling of surface area available for resource uptake, from which non-growth metabolism (referred to as catabolism by von Bertalanffy^35^) is then subtracted to obtain growth. Therefore, we hereafter refer to ‘anabolism’ as ‘biosynthesis potential’. The $K{m}^{B}$ term on the right-hand side of Eq. (1) represents resource consumption by tissues and is often proposed to scale in proportion to body mass^35^, i.e.$\,B=1$, though we will discuss potential causes of deviation from this value later.

A common assumption imposed on the VBGF is isomorphic scaling of biosynthesis potential, corresponding to growth without change in body shape, represented by the commonly chosen Euclidean value of $\frac{2}{3}\,$ for the mass-scaling exponent,$\,A$. This assumption is widely imposed despite recognition from von Bertalanffy of the potential range of values for $A$, for example, rod-like bacteria that grow in one-dimension of length ($A=1$), with volume increasing proportionally to length and to surface area for resource uptake^35^.

The Schnute model is a four-parameter growth model developed by Schnute^36^ often applied in aquaculture research^37,38^. The Schnute model has been proposed as superior to the VBGF for modelling growth of aquaculture species including the spotted rose snapper^39^, *Lutjanus guttatus*, and turbot^40^, *Scophthalmus maximus*. However, comparisons made between the Schnute model and the VBGF often apply the common parameterisation of $\frac{2}{3}$ scaling of parameter $A$ (Eq. (1))^40^, which limits the range of growth curves that can be captured. Additionally, Yuancai, Maraques & Macedo^41^ show through analytical transformation, that the Schnute model and the generalised VBGF (Eq. (1)) can be formally equivalent despite having different function forms and parameters: the two models gave the same growth predictions for stand density of *Eucalyptus grandis*. Therefore, by considering the flexibility of the VBGF a wide range of growth types can be captured and accurate predictions of growth can be achieved.

Restriction in the parameterisation of the mass-scaling of biosynthesis potential is also present in the Gompertz model^42^ which has been used to model growth of plants, birds, fish, mammals, tumour cells and bacteria^43^. Like the VBGF, the Gompertz model is also part of the Richards growth model family^19^ where it is a special case of both the VBGF and Richards model where a complementary limit arises when $A\,\to {1}^{-}$, where $K(A-\,1)$ is fixed^19^^.^ As the Gompertz model is achieved by calculating the body-size scaling of biosynthesis potential as a limit $(A\,\to {1}^{-})$ it assumes an exponential decline in absolute growth rate with body size, making it inappropriate for taxa displaying other growth types that range from isomorphic to supra-exponential. For example, during ontogeny thaliacean organisms, such as salps and doliolids^44^, exhibit increasing relative growth rate (RGR), the rate of body mass increase per unit mass per unit time, and thus have potential for supra-exponential growth.

Other well-known models with the same mathematical structure as the VBGF include the Dynamic Energy Budget (DEB) and the ontogenetic growth model (OGM), an extension of the ‘West, Brown and Enquist’ (WBE) model for metabolic scaling^45^, which has been developed and improved over time^46–48^. The OGM predicts the rate of energy devoted to growth is equal to the rate of assimilation of metabolic energy (the ‘anabolic’ term) minus the rate of energy allocated to maintenance (the ‘catabolic’ term). Although the mathematical structure is the same as the VBGF (Eq. (1)) the mechanism of growth varies. The OGM assumes a mass-scaling exponent of biosynthesis potential^48^ (assimilation) of $\frac{3}{4}\,$. As a result, application of the OGM to taxa with differing mass-scaling of resource supply is likely to result in poor-fitting growth curves and inappropriate predictions. Further, Hirst & Forster^49^ found poor fit of the WBE to marine invertebrate growth data due to overestimating body size early in ontogeny and underestimating later in ontogeny. We suggest that parsimonious versions of the VBGF may provide better fits, and incorporate more biologically meaningful parameters, than some other simple equations, such as the logistic model. The logistic model^50^ is regarded as the simplest of sigmoidal growth models with its symmetry about the point of inflection as given by the parameterisation^51^ ${L}_{t}=\frac{{L}_{\infty }}{1+{e}^{-c(t-1)}}$ . Shi *et al*.^52^ compared the performance of the OGM with the logistic model and a generalised VBGF given by: ${L}_{t}={L}_{\infty }{[1-\exp (-KD(t-{t}_{0}))]}^{1/D}$ where the mass-scaling exponent of biosynthesis potential $(A)$ ranges between 0.5 and 1. Based on Akaike Information Criterion (AIC) scores, the logistic model was found to be best fit for late-larval stage empirical growth data for three fish species. However, for all cases the value for $A$ for the VBGF was 1.0, suggesting that more parsimonious models such as the Gompertz or Exponential model may better fit the data where $A\,\to {1}^{-}\,$and $A=1$, respectively. Shi *et al*.^52^ argue that using a generalised version of the VBGF results in poor predictions of parameters, $K$ and ${t}_{0},$ but this may be resolved by applying the Gompertz or Exponential parameterisation of the VBGF. Additionally, it is unknown what a “good” prediction of ${t}_{0}$ in the generalised VBGF is, considering that ${t}_{0}$ is a mathematical artefact representing time at zero body mass and the biological interpretation of $K$ is debatable^53^. Furthermore, the authors determine goodness of fit of these models through use of R-squared, a method which is inappropriate for non-linear models^54,55^.

Despite the numerous debated biological mechanisms underpinning growth models, as discussed above, the VBGF (Eq. (1)) often prevails as a mathematical growth function, which can be parameterised in many ways to capture variation in RGR. Recent studies have highlighted growth curve diversity through the variation in the mass-scaling exponent of biosynthesis potential, $A$. Insects, for example, seldom grow isomorphically; instead, mass often scales almost in proportion to surface area, and the growth curve is near-exponential^56^. Thus it can be predicted that $\frac{2}{3} < A < 1$ for insect growth. Maino and Kearney^57^ found support for this hypothesis, with reported values of *A* between $\frac{3}{4}$ and 1 for the mass-scaling exponent of consumption and assimilation in 41 insect species. In addition, if oxygen uptake at rest is considered to be proportional to biosynthesis potential (as oxygen fuels both growth and non-growth, even at rest^58^), estimates of values of A may be derived from the mass-scaling of resting or routine metabolic rates. Thus, Killen *et al*.^59^ report values between $\frac{2}{3}$ and 1 for the body size scaling of resting metabolic rate for 89 species of teleost fish. The lack of universality in the mass-scaling of biosynthesis potential, if assumed to be proportional to routine metabolic rate, has also been highlighted within invertebrate species, which display a diverse range in the mass-scaling of oxygen consumption^60–62^. If the mass-scaling of metabolic rate does not hold universally it is suggestive that neither does the mass-scaling of growth, since growth is fuelled by metabolism (albeit only a component of the total respiration rate may relate to the costs of biosynthesis potential).

The above arguments highlight that when fitting growth curves to empirical data, a single fixed value or limit, for the body mass-scaling exponent of biosynthesis potential is unlikely to hold universally. Therefore, it is proposed that growth-curve fitting methods should not pre-determine this exponent, but instead allow for and test for all plausible possibilities. The importance of applying a multimodel approach to fitting growth curves has been shown by Reynaga-Franco *et al*.^38^ where different growth models were favoured by AIC for *Crassostrea gigas* raised under identical conditions. Evidence^62,63^ suggests most variation among diverse aquatic taxa relates to scaling of surface area, and hence to the scaling of biosynthesis potential ($Hm$). By contrast, we argue that the scaling of non-growth metabolism or catabolism ($Km$) varies less among organisms, and as assumed by von Bertalanffy^35^ and Kooijman^64,65^, scales approximately linearly with body mass where $B=1$. We recognise that this assumption is contentious and may require modification for certain taxa, where catabolism (or maintenance) does not necessarily scale in proportion to body volume, such as when the proportion of body composition taken up by non-metabolising fat reserve increases during ontogeny, as reported in some insects^57^.

Previous work by Ohnishi *et al*.^66^ addressed the need to allow mass-scaling exponents to vary when applying the VBGF to organisms. These authors developed a standardised form of the VBGF which allowed variation in both exponents $A$ and $B$. However, the derivation of their solution effectively ensures that the value of exponent $A$ cannot exceed exponent $B$. Consequently, if we are to fix $B=1$, we cannot estimate values of $A$ greater than 1. This becomes problematic when organisms have supra-exponential growth ($A > 1$) such as in thaliaceans, as discussed above. In addition, Ohnishi *et al*. do not give methods for calculating confidence intervals or comparing estimates of exponent $A$ to obtain a best-fit value for an organism.

Growth rate has been shown to correlate with many life-history traits, such as fecundity and lifespan for numerous taxa including fish^67,68^, reptiles^69^, arthropods^70,71^, mammals^72,73^ and tetrapods^74^, making it a key determinant of organism fitness^75^. Therefore, our aim is to improve the flexibility and applicability of current growth-curve fitting methods by offering a new framework, based on the widely known VBGF (Eq. 1), that allows for diverse growth types (including both isomorphic and non-isomorphic) by applying a set of re-parameterisations that allow variation in the mass-scaling of biosynthesis potential. Marine invertebrates display diverse variation in the mass-scaling of growth and metabolic rate^61,62,76^ and thus provide an ideal group to test the applicability of this framework. Further, it has been shown by Glazier^76^ that pelagic and benthic invertebrates display marked variation in their metabolic mass-scaling relationships, with pelagic species having significantly greater metabolic mass-scaling exponents than benthic species. By exploring both open-water and bottom-dwelling invertebrate species we are able to capture potential diversity in growth rate that may be attributed by differences in lifestyle and environmental conditions.

## Materials and methods

### Developing candidate growth models

The solution^19^ to the original VBGF (Eq. (1)) when $B=1$ is:

2 $$\begin{array}{c}m={m}_{0}{\left\{\frac{1-(1-Z)\exp (K(A-1)(t-{t}_{0}))}{Z}\right\}}^{-\frac{1}{A-1}}\end{array}$$

where ${m}_{0}$ represents mass $m$ at time ${t}_{0}$ (time at birth/ hatch). The mass-scaling exponent for biosynthesis potential is given by $A$ and the rate at which final mass is reached is represented by parameter $K$. Parameter $Z={\left(\frac{{m}_{\infty }}{{m}_{0}}\right)}^{A-1}$, where ${m}_{\infty }={\left(\frac{H}{K}\right)}^{1/(1-A)}$, has no simple biological interpretation. While Eq. (2) represents a valid solution for all $A > 0$, it is not the most suitable form for fitting to data because of collinearity of parameters, and because the expression is singular when $A=1$. We find that different parameterisations are appropriate for the parameter $A$, corresponding to the Pure Isomorphy model (VBGF) and four nested non-isomorphic growth models: Exponential, Gompertz, Generalised-VBGF and Supra-exponential. These five parameterisations represent different categories of relative growth rate (RGR) (i.e. the body mass increase per unit mass per unit time)^77^, including constant RGR over time (Exponential model), decreasing RGR over time (Gompertz, Generalised-VBGF and Pure Isomorphy models) and increasing RGR over time (Supra-exponential model). For full derivation of Eq. (2) and further detail of the five parameterisations see Supplementary information.

### Parameterisation of the Exponential model

When $A=1$ relative growth rate is constant and growth is purely exponential, which yields the solution

3 $$m={m}_{0}\,\exp (k(t-{t}_{0}))$$

Where $k=H-K$. Firstly, we fit this model by setting ${m}_{0}$ as the mass at the first time point. This solution involves fitting just one parameter, $k$. Parameter $k$ is estimated iteratively, after inputting the reasonable start value of 0.1. This estimate is subsequently used as a starting value, along with ${m}_{0}$ as the mass at the first time point, for the subsequent model run where we fit parameter ${m}_{0}$.

### Parameterisation of the Gompertz model

The Gompertz model is a generalisation of the exponential model and a special case of the General-VBGF^35^ where RGR decreases over time as the exponent of biosynthesis potential, $A$, approaches limit$\,A\to {1}^{-}$, represented by a second parameterisation $(b,\,k)$ (see Supplementary information for derivation):

4 $$\mathop{\mathrm{lim}}\limits_{A\to {1}^{-}}m={m}_{0}\exp [\,-\,b(\exp (-k(t-{t}_{0})-1))]$$

When parameter ${m}_{0}$ is initially fixed and ${t}_{0}$ is known, this involves estimating two parameters: $b\,$and$\,K$. Starting values for $k$ are taken from the estimates of the exponential model, and the starting value for $b$ is chosen so that the asymptotic mass predicted by the model is twice the largest mass in the data. The justification is that the starting value must be larger than the largest mass in the data set for the fitting to work. If this value is too much larger, then the fit will be indistinguishable from an exponential solution and so the fitting will struggle to identify the asymptote, which makes a factor of two a good compromise to ensure the inflection in the model is tested against the data.

### Parameterisation of the Generalised-VBGF

The Generalised-VBGF allows for non-isomorphic growth where RGR decreases over time where the mass-scaling exponent $A$ can hold a value between 0 and 1. We encountered problems when fitting the model by varying the parameters $A$, $Z$, and $K$, because of strong collinearity between $A$ and $K$, and because of numerical roundoff errors when $Z$ was close to 1. We therefore fitted the model by varying the parameters $(A,\,f,\,k)$ where $k=(A-1)K$ and $f=1-Z$. In terms of these parameters, Eq. (2) can be written as:

5 $$m={m}_{0}{\left\{\frac{1-f\exp (-k(t-{t}_{0}))}{1-f}\right\}}^{-\frac{1}{A-1}}$$

The parameter range that represents biological growth is $0 < f < 1$, $0 < A < 1$, $k > 0$.

When $A$ is close to 1 we expect $k$ to be similar to its value in the Gompertz model and so we apply the estimates from the Gompertz model as starting values for the Generalised-VBGF. The initial values for the other parameters are given by:

6 $$(1-A)=\,\min \left({a}_{max},\,\frac{{f}_{max}}{\max (b)}\right)$$

7 $$f=(1-A)\max (b)$$

where ${a}_{max},{f}_{max}$ are chosen numbers between 0 and 1, and ${\rm{\max }}(b)$ is the largest fitted value of $b$ (amongst all individuals of the species under consideration) from the Gompertz model. This ensures that the initial values of $f$ and $A$ are in the biologically relevant range.

### Parameterisation of the Pure Isomorphy model

Under three-dimensional Euclidean geometry, growth that is purely isomorphic is represented by the fixed value of $\,\frac{2}{3}\,$ for the mass-scaling exponent,$\,A$, and hence is a reduced version of the Generalised-VBGF where $A\,=\frac{2}{3}$. This means only two parameters are estimated: $f$ and$\,K$ from starting values obtained from the estimates given by the Generalised-VBGF.

### Parameterisation of the Supra-exponential model

The case $A > 1$ occurs when RGR increases over time and corresponds supra-exponential growth, but the model exhibits biologically unrealistic behaviour, such as infinite mass, unless the parameter values are chosen with care. To avoid this, the optimiser varied parameters $Z$, $\alpha$, and $s$, where $\alpha =\frac{1}{A}$, $s=-({t}_{max}\,-\,{t}_{0})\frac{K(A-1)}{\log (1-Z)}$ and ${t}_{max}$ is the largest value of $t$ in the data set for the individual in question. The full biologically relevant parameter space corresponds to each of $Z$, $\alpha$, and $s$ being constrained to lie between 0 and 1. To give the original biological parameters we invert the estimates by the transformations:

7 $${m}_{\infty }={m}_{0}{Z}^{\frac{1}{A-1}}$$

8 $$A=\frac{1}{\alpha }$$

9 $$K=-\frac{s\,\log (1-Z)}{(A-1)({t}_{max}-{t}_{0})}$$

Candidate starting values for these parameters are chosen so that the solution is close to the fitted exponential model. To achieve this, we choose $Z\,$to be small, $A\,$ to be just greater than 1, and $K=kZ$ (where $k\,$is taken from the exponential model fit). We then use the above formulae to compute the corresponding values of $\alpha$, and $s$.

## Fitting and assessing candidate growth models

### Model fitting in R

The five candidate models were fitted to empirical mass-time data with log least-squares method of optimisation by using the general-purpose optimisation function *optim()* in R (v3.5.0) (see Supplementary R code and Supplementary appendix I for user guide). This function was chosen for its robust method of applying Nelder-Mead algorithms. Since optim() does not allow constrained Nelder-Mead optimisation, biological parameters were transformed (using a log or logit transform) so the biologically meaningful range corresponded to $(-\infty ,\infty )$ in the space explored by optim().

Optimisation initially fitted the models with the ${m}_{0}$ parameter fixed at the first empirical mass value. Parameter estimates gained from this optimisation were consequently used as starting parameters for optimisation where the ${m}_{0}$ parameter was estimated. It is often unrealistic that the first recorded mass value is the precise mass at time zero (at birth or hatch) and so only the optimised parameter estimates for model fitting where ${m}_{0}$ was estimated were used in subsequent analysis. Hence, the purpose of carrying out optimisation where ${m}_{0}$ is fixed at the first empirical mass value was to produce reasonable starting values for *optim()*.

Log least-squares fitting was chosen over least-squares because it allows for more weighting of error at smaller mass values. This comes from the reasoning that it is biologically realistic to assume fluctuations in growth rate between individuals are proportional to body size, i.e. individuals will grow similarly initially but display more variation in size (mass) later in life. To determine the best fitting value for the mass-scaling exponent of biosynthesis, $A$, the model with the most negative log likelihood value was taken as the best fit model. Confidence intervals for parameter *A* were constructed using profile likelihood in R (v3.5.0) (see Supplementary appendix I for user guide on executing the relevant R code). We use a purely likelihood-based approach, rather than the Akaike Information Criterion, because our focus is on providing a confidence interval for the parameter *A* rather than in selecting which single model (i.e. value of *A*) to use for forecasting. The 95% confidence intervals show the range of values of *A* that would not be rejected as a null model, and hence are consistent with the data.

## The data set

Aquatic invertebrates assimilate resources through different body surfaces, for example, integument and/or gills for oxygen uptake. Differences in environmental conditions (e.g. predation) that exist between benthic and pelagic habitats of aquatic invertebrates may affect the mass-scaling of an organism’s uptake of resources. For example, high predation risk throughout ontogeny in the sunlit epipelagic zone, which lacks refuges from predators, may lead to the evolution of steeper mass-scaling of resource uptake, compared with more benthic conditions where invertebrates can reduce predation risk by finding refuge^78–80^. The diversity in the mass-scaling of biosynthesis potential makes benthic and pelagic invertebrate species two ideal groups to explore variation in the mass-scaling exponent of biosynthesis potential $(A)$ when fitting the VBGF.

Published ontogenetic mass-at-age data were collected for seven pelagic and five benthic invertebrate species using Web of Knowledge. Search terms included “growth AND pelagic AND (lab* OR cultur* OR ontogen* OR development*)” for pelagic species and “growth AND benthic AND (lab* OR cultur* OR ontogen* OR development*)” for benthic species. We chose species based on availability of growth data that conform to the specific requirements described below. To provide a diverse sample of growth curve fits to empirical data, we chose species comprising both gelatinous and non-gelatinous zooplankton across four phyla: Arthropoda, Cnidaria, Chordata and Mollusca. Species were considered pelagic or benthic based on the zone inhabited by the developmental stage in which growth data was obtained from. For example, for many adult benthic invertebrates the larval stage occurs in the pelagic zone, e.g. many decapod species that occur in the pelagic zone during their zoeal stage before migrating to their benthic habitat. The species used in analysis were as follows. Pelagic: *Daphnia magna* (Branchiopoda)^81^ *Pelagia noctiluca* (Scyphozoa)^82^, *Euphausia pacifica* (Euphausiacea)^83^, *Oikopleura dioica* (Appendicularia)^84^, *Aurelia aurita* (Scyphozoa)^85^, *Cyanea capillata* (Scyphozoa)^86^ and *Crassostrea gigas* (Bivalvia)^87^. Benthic: *Mytilus edulis* (Bivalvia)^88^, *Sepia officinalis* (Cephalopoda)^89^, *Echinogammarus marinus* (Amphipoda)^90^, *Cherax quadricarinatus* (Decapoda)^91^ and *Petrarctus demani* (Decapoda)^92^. Species identities were checked using the World Register of Marine Species (WoRMS) to ensure accepted names were used.

When required, data were extracted from graphs using the software WebPlotDigitizer (Rohatgi, 2017). Data were accepted if collected under controlled and constant environments; field data were therefore excluded. Mass data selected were from time at hatch until reproductive maturity and did not include data from mature animals. We used the time of reproductive maturity determined by the authors themselves, or, when this was unavailable, an approximate age at maturity at the given temperature was obtained from the scientific literature. Data for *C. gigas*, *A. aurita* were from pelagic larvae or juveniles and *M. edulis* data were from benthic juveniles, and did not include growth data up to maturity (incomplete juvenile development) due to lack of available data that conform to our data requirements. Therefore, we recognise that for these three species utilising data across larger parts of life history may result in different model fits. Our data requirements were as follows. Growth data were not collected when conditions included starvation, predation or toxin treatments, or temperatures/salinities beyond the normal range encountered by the species in its natural setting. Mass type (either dry, ash-free or wet), treatments, culture conditions, developmental stages, sex and site of origin were also recorded. If only length data were available, we applied published length-mass conversion equations for a given species.

## Results

### Comparison of models across species

**Figure 1:**
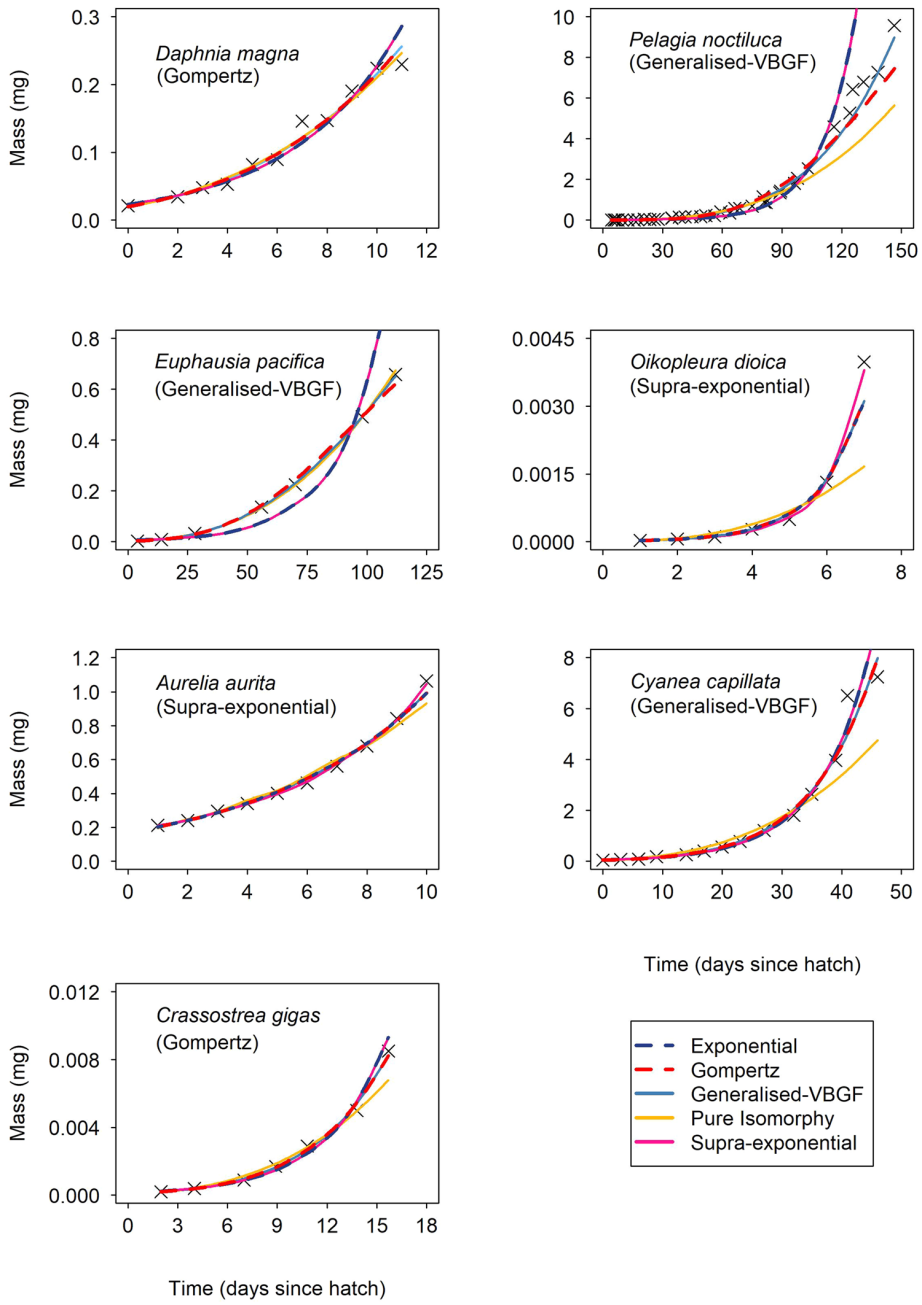
Model fits for the five von Bertalanffy growth function (VBGF) (Eq. 1) parameterisations (Eq. 1) for empirical mass versus time data for seven species of pelagic invertebrates with the best fit model given in brackets. From top left: *Daphnia magna* (Gompertz), *Pelagia noctiluca* (Generalised-VBGF), *Euphausia pacifica* (Generalised-VBGF), *Oikopleura dioica* (Supra-exponential), *Aurelia aurita* (Supra-exponential), *Cyanea capillata* (Generalised-VBGF) and *Crassostrea gigas* larvae (Gompertz).

**Figure 2:**
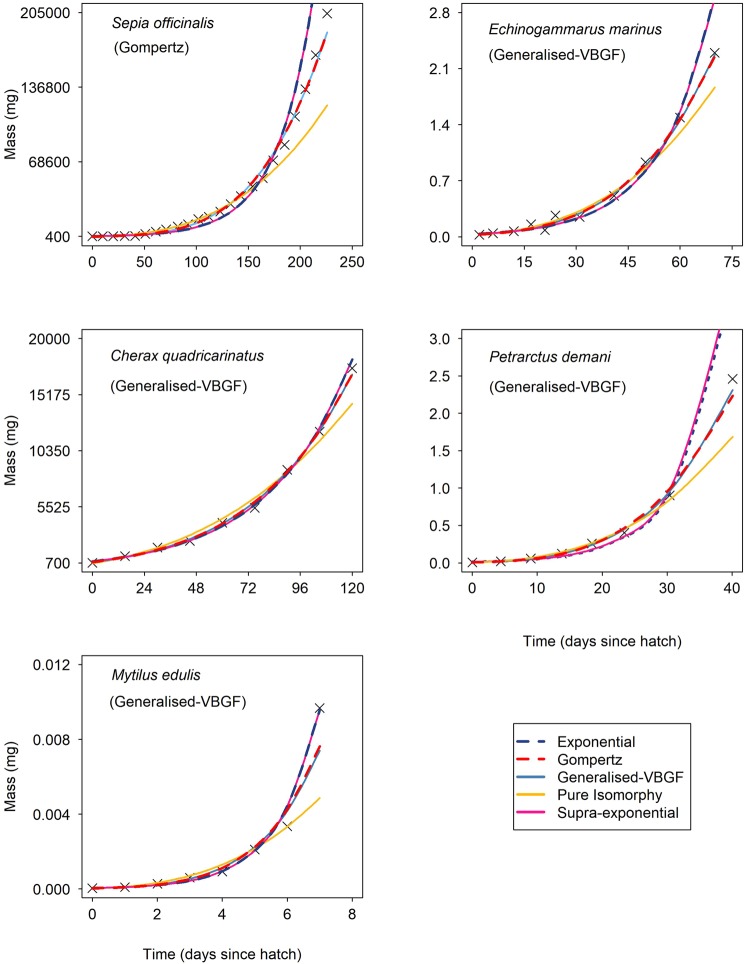
Model fits for the five von Bertalanffy growth function (VBGF) (Eq. 1) parameterisations for empirical mass versus time data for five species of benthic invertebrates with the best fit model given in brackets. From top left: *Sepia officinalis* (Gompertz), *Echinogammarus marinus* (Gompertz), *Cherax quadricarinatus* (Exponential), *Petrarctus demani* (Generalised-VBGF) and *Mytilus edulis* (Generalised-VBGF).

The negative log likelihood values for the five candidate re-parameterisations of the von Bertalanffy Growth Function (VBGF) showed that there was no universal agreement in best-fitting VBGF model across the twelve pelagic and benthic invertebrate species with a range of best-fitting values for the mass-scaling exponent of biosynthesis potential, $A$, between 0.76 and 1.22 (Table 1) (see Supplementary appendix I Table S1 for negative log likelihood values). Both pelagic and benthic species displayed the same mixture of best-fitting models including the Generalised-VBGF, Gompertz and the Supra-exponential model (Figs. 1 and 2). The Generalised-VBGF was found to be the best fit for 58% (7 out of 12) of species, followed by the Gompertz (25%) and Supra-exponential (17%) model (Table 1). The two models where parameter $A$ remains fixed, the Exponential and Pure Isomorphy model, were not found to be the best fit for any species.

**Table 1:** The best-fitting values for the mass-scaling exponent for biosynthesis potential, $A$, as determined by the most negative log-likelihood between the five parameterisations of the VBGF: Exponential, Gompertz, Generalised-VBGF, Pure Isomorphy and Supra-exponential for empirical mass versus time data for twelve pelagic and benthic invertebrate species. The zone (pelagic or benthic) represents the zone inhabited during the development phase in which growth data was obtained for. The number of datapoints is represented by N. The 95% confidence intervals for parameter $A$ were calculated using profile likelihood.

Habitat	Zone	Phylum	Class	Species	N	Best fit model	*d.f*.	${\boldsymbol{A}}$ estimate	95% confidence intervals
Freshwater	Pelagic	Arthropoda	Branchiopoda	*Daphnia magna*	11	VBGF-Gompertz	7	1.0	0.58 – 1
Marine	Pelagic	Arthropoda	Malacostraca	*Euphausia pacifica*	7	Generalised-VBGF	2	0.79	0.68 – 0.91
Marine	Pelagic	Cnidaria	Scyphozoa	*Pelagia noctiluca*	39	Generalised-VBGF	34	0.76	0.73 – 0.78
Marine	Pelagic	Chordata	Appendicularia	*Oikopleura dioica*	7	VBGF-Supra-exponential	2	1.12	1.06 – 1.16
Marine	Pelagic	Cnidaria	Scyphozoa	*Aurelia aurita*	10	VBGF-Supra-exponential	5	1.22	1.21 – 1.32
Marine	Pelagic	Cnidaria	Scyphozoa	*Cyanea capillata*	14	Generalised-VBGF	9	0.92	0.88 – 0.96
Marine	Pelagic	Mollusca	Bivalvia	*Crassostrea gigas*	7	VBGF-Gompertz	3	1	0.80 – 1
Marine	Benthic	Arthropoda	Malacostraca	*Echinogammarus marinus*	11	Generalised-VBGF	7	0.79	0.64 – 0.93
Freshwater	Benthic	Arthropoda	Malacostraca	*Cherax quadricarinatus*	9	Generalised-VBGF	4	0.89	0.81 – 0.95
Marine	Benthic	Arthropoda	Malacostraca	*Petrarctus demani*	8	Generalised-VBGF	3	0.79	0.76 – 0.93
Marine	Benthic	Mollusca	Bivalvia	*Mytilus edulis*	8	Generalised-VBGF	3	0.87	0.79 – 0.95
Marine	Benthic	Mollusca	Cephalopoda	*Sepia officinalis*	23	VBGF-Gompertz	19	1.0	0.80 – 1

### Comparison of models across taxa

Across the arthropods the Generalised-VBGF was the best fit for all four malacostracan species (Table 1), whereas the branchiopod *Daphnia magna* had a growth trajectory best fit by the Gompertz model (Fig. 1). Cnidarian species *Pelagia noctiluca* (Fig. 1) and *Cyanea capillata* (Fig. 2) both displayed decreasing RGR with the Generalised-VBGF model (where $A$ = 0.76 and 0.92, respectively), whereas, during an incomplete juvenile development, the cnidarian *Aurelia aurita* (Fig. 2) displayed increasing RGR with the Supra-exponential model as the best fit ($A$ = 1.22) (Table 1). The appendicularian, *Oikopleura dioica*, also displayed supra-exponential growth where $A$ = 1.12 (Fig. 1). Across the molluscs, there was no universal agreement in best-fitting model for the incomplete developmental growth of the two bivalve species, *Mytilus edulis* and *Crassostrea gigas* agreeing with the Generalised-VBGF and the Gompertz model, respectively and the benthic cephalopod *Sepia officinalis* agreeing with the Gompertz model (Table 1).

## Discussion

A range of values for the mass-scaling exponent of biosynthesis potential, $A$, $(0.72 < A\le 1.22)$ (Table 1) highlights the diversity of growth curves amongst species (Figs. 1 and 2). This proposed framework for fitting growth curves provides improved predictions of growth and increased model validity for species displaying growth curves that differ from commonly fixed values of the mass-scaling of synthesis such as $\frac{2}{3}$ (isomorphic growth) or 1 (pure exponential growth). This includes two cases of supra-exponential growth (where $A > 1$) found in the appendicularian *Oikopleura dioica* (Fig. 1) and during part of juvenile development of the scyphozoan *Aurelia aurita* (Fig. 2) (Table 1). Widespread diversity in the mass-scaling of biosynthesis potential highlights the range of growth curves present amongst organisms. This brings into question current methods of growth curve-fitting which impose a fixed value, limit or range for exponent $A$ that are unable to capture variation in the mass-scaling of biosynthesis potential, and consequently growth rate.

Both pelagic and benthic species displayed variation in the best-fitting model, suggesting that there is no general difference in pattern of growth between pelagic and benthic species or ontogenetic phases, although a larger sample would be required to test this more definitively. Generally, there was no trend between best-fitting model and taxonomic group, except for the malacostracan crustacean growth curves, which all agreed with the Generalised-VBGF (Table 1). The Generalised-VBGF is a flexible model, allowing$\,A$ to vary between 0 and 1, so even though all malacostracan species display the same best-fitting model they show diversity in exponent $A$. This lack of consensus in the best-fitting growth model within taxonomic groups in this study indicates a potentially problematic issue with applying a single growth model when studying specific taxonomic groups.

Gaining accurate predictions of exponent $A$ can aid biological understanding and open up new hypotheses. For example, the steep mass-scaling ($A=1.12$) of *O.dioica* during ontogenetic growth prompts suggestions about the selective effects on growth of mortality risk in an open-water environment. With no refuges from predators, rapid sustained uptake of resources may be required to reach maturity fast before being consumed^79,80^. The scyphozoan *Pelagia noctiluca* also exists within a high-mortality pelagic environment but instead exhibits a shallower mass-scaling of biosynthesis potential ($A=0.76$). This difference in exponent can prompt hypotheses about selective differences in mortality risks, including whether mortality reduces as size increases, or whether energy is invested into functions other than growth such as locomotion and/or buoyancy mechanisms. Furthermore, variation in the mass-scaling of biosynthesis potential was also present amongst benthic species (Table 1). For example, the common cuttlefish, *Sepia officinalis*, exhibits rapid exponential growth where relative growth rate (RGR) is constant ($A\,=\,1$) (Fig. 2), whereas the amphipod *Echinogammarus marinus* displays decreasing RGR where $A\,=0.79$ (Fig. 2). Despite partial covering of sand/seaweed, the predation risk for *S.officinalis* may be high considering the lack of parental care of eggs and high rates of cannibalism^93^. The relatively short lifespan of one to two years for *S.officinalis*^94^ supports the idea that sustained rapid growth is required to reach maturity before dying. In contrast, *E.marinus* lives sheltered under algae, mud and/or rocks and exhibits egg development fully within the brood pouch^90^. These features are indicative of low mortality risk throughout development, suggesting that gains in survival may accrue from investing in survival at the expense of sustained rapid feeding and exponential growth. Thus, fitting growth curves under this proposed framework helps formulate specific testable hypotheses about the selective effects of an organism’s ecology on their growth.

The lack of universal agreement in the best-fitting growth model suggests applying a single parameterisation is not necessarily the best method of fitting growth curves to data. Instead, using a framework based on a set of parameterisations of a prevailing mathematical function increases flexibility (by allowing for variation in $A$). Flexibility enables us to find the best-fitting model with reliable predictions of growth and capture variation in growth rate, i.e. isomorphic and non-isomorphic growth. Ultimately, this framework enhances model applicability to a wider range of taxa. To further test and explore this framework, future work should focus on testing the validity of the $B=1$ assumption for the mass-scaling of maintenance often made in the VBGF. It was assumed by von Bertalanffy^35^ that $B=1$ on the basis that maintenance costs are approximately proportional to body mass. However, for some organisms, body mass composition can change throughout ontogeny, for example, insects have been shown to have increasing energy reserves (non-metabolising body mass) with age, which results in reduced mass-specific maintenance costs^57^. Therefore, we recognise the need for flexibility in parameter $B$ for certain animal groups where maintenance does not scale in proportion to body mass.

To achieve accurate predictions of growth rates, the pattern of growth must be accurately captured by the growth model. The common $\frac{2}{3}$ parameterisation (Pure Isomorphy model) of the VBGF captures sigmoidal growth patterns whereby growth rate declines over time^35^. For organisms where mass-specific growth rate is maintained (exponential growth) or increased (supra-exponential growth) a sigmoidal growth function will predict lower than expected mass-specific rates of growth over time – resulting in poor predictions of growth. Our results show that while the five VBGF models can produce almost indistinguishable growth predictions in some cases, for example the Gompertz and Generalised-VBGF model for larval *Crassostrea gigas* (Fig. 1), over the twelve species (Figs. 1 and 2) the five models can show great differences in growth predictions for given data. For example, applying the Pure Isomorphy model to *S.officinalis* (Fig. 2) would underestimate late juvenile growth whereas the Supra-exponential and Exponential models would overestimate this growth.

Instead, the proposed growth curve fitting procedure for the five parameterisations of the VBGF allows the optimal value for exponent $A$ to be found which results in the most accurate predictions of growth obtained by the VBGF. Hence, this procedure offers application of the VBGF to a wider range of taxa such as marine invertebrates which have previously poorly fitted the VBGF^49^. Modelling growth of marine invertebrates has proved difficult, for example, in sea cucumbers owing to their naturally flaccid bodies and ability to shrink in size (degrow)^95^, but accurate growth predictions are key to understanding how well species may survive in specific environmental conditions.

Extensive and successful use of the VBGF occurs for numerous fish species to aid the understanding of growth in relation to reproduction^68^, fishing mortality^96^ and environmental temperature^97^, all of which are relevant to the sustainability of aquaculture. By applying this growth curve-fitting framework, we extend the range of taxa to which the VBGF (Eq. (1)) can be applied and hence to a wider range of ecological issues, such as the sustainability of marine invertebrate aquaculture.

## Supplementary information

Supplementary Information.

## Data Availability

Code to reproduce the fitting of the five VBGF parameterisations can be found at (https://github.com/lauraleemoore/Growth-curve-fitting).
