# Supplementary material for: A new framework for growth curve fitting based on the von Bertalanffy Growth Function
Source: Sci Rep. 2020 May 14;10:7953. doi: 10.1038/s41598-020-64839-y (PMC7224396; doi:10.1038/s41598-020-64839-y)
Supplement: Supplementary file 1 — Supplementary Information. [file 41598_2020_64839_MOESM1_ESM.pdf]

Supplementary information for: A new framework for growth curve fitting based on the von Bertalanffy Growth Function.

Laura Lee[1*], David Atkinson[1], Andrew G. Hirst[2,3], Stephen J. Cornell[1]

[1]Department of Evolution, Ecology and Behaviour, University of Liverpool, UK [2]School of Environmental Sciences, University of Liverpool, UK; [3]Centre for Ocean Life, DTU Aqua, Technical University of Denmark, Lyngby, Denmark

Supplementary Methods. Mathematical derivations for the five von Bertalanffy Parameterisations

1 General solution

The generalised von Bertalanffy equation (GvBE) is

$$\frac{dm}{dt} = Hm^A - Km^B. \tag{1}$$

A solution for general A and B is given in Ohnishi *et al.* (2014) of the incomplete Beta function, but this turned out not to be ideal for our purposes because we could not find an implementation in R that could evaluate this special function robustly for the parameter range explored by the fitting (in particular, when A was close to 1). However, when $B = 1$ the equation can be solved in terms of elementary functions (Richards 1959). We first make the change of variable $m = x^b$, so that

$$\begin{aligned} bx^{b-1}\frac{dx}{dt} &= Hx^{bA} - Kx^b \\ \Rightarrow \frac{dx}{dt} &= \frac{H}{b}x^{bA+1-b} - \frac{K}{b}x. \end{aligned}$$

If we choose b such that $bA + 1 - b = 0 \Rightarrow b = \frac{1}{1-A}$, then x satisfies the linear ODE

$$\frac{dx}{dt} = H(1-A) - K(1-A)x,$$

whose general solution is $x = \frac{H}{K} + c\exp(-K(1-A)x)$ (where c is a constant). If the initial contitions are $m = m_0 = x_0^b$ when $t = t_0$,then

$$\begin{aligned} x &= x_\infty + (x_0 - x_\infty)\exp(-K(1-A)x) \\ \Rightarrow m &= m_0 \left\{ \frac{[1-(1-Z)\exp(-K(1-A)(t-t_0))]}{Z} \right\}^{-\frac{1}{A-1}}, \end{aligned} \tag{2}$$

where $x_\infty = H/K$ and

$$Z = \left(\frac{m_\infty}{m_0}\right)^{A-1},$$

with $m_\infty = x_\infty^{1/(1-A)}$

2 Exponential, $A = 1$.

When $A = 1$ the GvBE becomes

$$\frac{dm}{dt} = (H - K)m,$$

which can be solved directly as $m = m_0 e^{(H-K)t}$. This solution can also be obtained from the general solution by taking the limit $A \to 1$ with K and H fixed.

3 Gompertz, $A \to 1^-$

A complementary limit arises when $A \to 1^-$, $K(A-1)$ fixed (Richards 1959). We first write,

$$\begin{aligned} m =& m_0 \left\{ \frac{[1 - (1-Z)\exp(K(A-1)(t-t_0))]}{Z} \right\}^{-\frac{1}{A-1}} \\ =& m_0 \frac{\left[1 - \left(\frac{1-Z}{1-A}\right) \frac{\exp(K(A-1)(t-t_0))}{y}\right]^y}{\exp\left(\frac{1-Z}{1-A}\right)}, \end{aligned}$$

where $y = -\frac{1}{A-1}$, If we take the limit

$$y \to \infty, \frac{(1-Z)}{1-A} = b, K(A-1) = -k,$$

where $b > 0$, $k > 0$ are constants, and use the relationship

$$\lim_{u \to \infty} \left(1 + \frac{x}{u}\right)^u = \exp(x),$$

then we find

$$\lim_{A \to 1^-} m = m_0 \exp\left[-b\left(\exp\left(-k(t-t_0)\right) - 1\right)\right].$$

Biologically relevant solutions occur in the parameter range $0 < b < \infty$, $0 < k < \infty$.

Biologically, this looks like a rather unusual limit for the original GvBE, since it implies (Richards 1959)

$$\begin{aligned} K &= ky \to \infty \\ H &= ZKm_0^{A-1} \\ &= (1-b)kym_0^{-\frac{1}{y}} \to \infty. \end{aligned}$$

Nevertheless, it represents a subspace of nonsingular solutions (2) of the GvBE, so we need to ensure that the model fitting algorithm is capable of accessing this limit.

To choose starting values for fitting the Gompertz model, we choose k to take the same value as the exponential. The parameter value b determines the asymptotic mass, as $\lim_{t\to\infty} m = m_0 \exp(b)$, so a plausible starting value is given by $m_\infty = 2m_{max}$, where m_{max} is the largest mass in the data, implying $b = \log(2m_{max}/m_0)$,.

4 Generalised growth model, $A < 1$

If we write $f = 1 - Z$, $k = K(1-A)$ then the solution (2) becomes

$$m = m_0 \left\{ \frac{[1 - f\exp(-k(t-t_0))]}{1-f} \right\}^{-\frac{1}{A-1}}.$$

For $0 < A < 1$, biologically relevant solutions represent the parameter range $0 < k < \infty$, $0 < f < 1$.

When choosing starting parameter values for the model fitting, we want to exploit the fitted values for the Gompertz model. Note that A is assumed not to differ within species, whereas b is fitted independently for each individual, so similarly f takes separate values for each individual in the species. If we set $f = b(1-A)$, then when $1 - A \ll 1$ this solution becomes

$$\begin{aligned} m &= m_0 \frac{\{1 - b(1-A)\exp(-k(t-t_0))\}^{-\frac{1}{A-1}}}{\{1-b(1-A)\}^{-\frac{1}{A-1}}} \\ &\approx m_0 \exp\left(b\left[\exp(-k(t-t_0)) - 1\right]\right), \end{aligned}$$

so for A close to 1 we expect the solution to be close to the Gompertz function with the same fitted values of b and k. However, using the Gompertz fitted value of b and an arbitrary starting value of A is dangerous because because $f = b(1-A)$ might exceed 1. Similarly, choosing an arbitrary starting value for f is also dangerous because $A = 1 - f/b$ would not be guaranteed to be positive. Instead, we choose the following starting values:

$$\begin{aligned} A &= 1 - \min\left(a_{max}, \frac{f_{max}}{\max(b)}\right) \\ f &= (1-A)b, \end{aligned}$$

where $\max(b)$ is the largest fitted value from the Gompertz model fitted to all the individuals of the same species, and a_{max} and f_{max} are chosen to be numbers bwetween 0 and 1. This then ensures that the initial values for all individuals are in the range $0 < A < 1$ and $0 < f < 1$.

5 Supra-exponential growth, $A > 1$

The GvBE (1), and its solution (2), can describe supra-exponential growth when $A > B = 1$ and H and K are both positive. However, the fixed point $m_\infty = (H/K)^{1/(1-A)}$ is now unstable (rather than stable, as for the case $A < 1$), so that if $m_0 < m_\infty$ then the organism shrinks to nothing rather than growing. Also, the solution has a singularity where $m \to \infty$ as $t \to t_*$, with

$$t_* = t_0 - \frac{1}{K(A-1)} \log\left(1 - \left(\frac{m_\infty}{m_0}\right)^{A-1}\right).$$

This means that the parameters need to be chosen with care so as to ensure that the solution does not cross into biologically inappropriate regimes, and so that the fitting is stable.

We define the following parameters

$$\begin{aligned} Z &= \left(\frac{m_\infty}{m_0}\right)^{\frac{1}{A-1}} \quad (0 < Z < 1) \\ s &= \frac{t_{max} - t_0}{t_* - t_0} \quad (0 < s < 1) \\ \alpha &= \frac{1}{A}(0 < \alpha < 1), \end{aligned}$$

where t_{max} is the largest value of t in the time series. The original biological parameters are given by

$$\begin{aligned} m_\infty &= m_0 Z^{\frac{1}{A-1}} \\ A &= \frac{1}{\alpha} \\ K &= -\frac{s \log(1-Z)}{(A-1)(t_{max} - t_0)}. \end{aligned}$$

Thus we see that the full range of biologically relevant parameters is given by keeping Z, s, α all in the range 0 to 1, since this ensures that $m_0 > m_\infty$, $1 < A < \infty$, and that t never exceeds t_*

When

$$
\begin{aligned}
m &= m_\infty \left\{1 - (1 - Z)\exp\left(K(A-1)(t-t_0)\right)\right\}^{-\frac{1}{A-1}} \\
&= m_\infty \left\{1 - (1 - Z)\left(1 + K(A-1)(t-t_0) + \dots\right)\right\}^{-\frac{1}{A-1}} \\
&= m_0 \left\{1 - \frac{(1-Z)}{Z} K(A-1)(t-t_0) + \dots\right\}^{-\frac{1}{A-1}} \\
&\to m_0 \exp\left(\frac{(1-Z)}{Z} K(t-t_0)\right). \text{ as } A \to 1
\end{aligned}
$$

Therefore, when $A \to 1^+$, with Z fixed and $\ll 1$, the solution is close to exponential growth with $k = K/Z$. We therefore choose starting values A slightly larger than 1, Z small, and then compute $K = Zk$, were k is the fitted value from the exponential model.

Supplementary appendix I. A user guide for executing the von Bertalanffy based growth modelling R code as proposed in:

A new framework for growth curve fitting based on the von Bertalanffy Growth Function.

Authors: Laura Lee[1*], David Atkinson[1], Andrew G. Hirst[2,3], Stephen J. Cornell[1]

[1]Department of Evolution, Ecology and Behaviour, University of Liverpool, UK

[2]School of Environmental Sciences, University of Liverpool, UK

[3]Centre for Ocean Life, DTU Aqua, Technical University of Denmark, Lyngby, Denmark

i. Data format requirements

For these models, growth data must be in terms of body mass and time. Datasets must numerically (from 1) distinguish between individuals of a species and between species. This must be labelled for each data entry point. Where cohort growth data for a species is being used then this should be labelled as a single individual. Therefore, datasets must have columns: mass, time, individual, species, for example:

species	individual	mass	time
1	1	5	0
1	1	10	2
1	1	15	4
1	1	20	6
1	2	7	0
1	2	12	2
1	2	15	4
1	2	21	6
2	1	2	0
2	1	4	2
2	1	7	4
2	1	10	6
3	1	1	0
3	1	3	2
3	1	6	4
3	1	8	6
3	1	12	8

For excel datasets, a single sheet must be used and saved as a Comma Delimited file (.csv).

ii. Running growth model R code

In R, the script “VBGFmodels_functions.R” must be completely run first to create all the five VBGF parameterisations and appropriate functions for model fitting in R. Next, the file “VBGFmodels.R” can be used to perform growth analysis on given growth data. For datasets with a single species use only the “unif.growth.mod” function. For datasets with multiple species run the “unif.growth.mod” function first and then the “unif.growth.mod.spec” function. After the function(s) have run, the growth analysis is carried out by simply inputting your data into the function(s):

```
yourgrowthresults < - unif.growth.mod(yourdata)
```

To save the model outputs use the save function in R:

```
save(yourgrowthresults, file="growthmodellingresults.Rdata")
```

This stored R file can then be reloaded into R using the load() function:

```
load("yourgrowthresults.Rdata")
```

iii. Calculating profile likelihood confidence intervals in R

To compute the profile likelihood 95% confidence intervals for parameter A, the two files are used: “Profile_likelihood_confidence_intervals_Alt1model.R” (which explores parameter $A < 1$) and “Profile_likelihood_confidence_intervals_Agt1model.R” (which explores parameter $A > 1$).

The 95% confidence interval for A is defined as the range of values for which the profile log likelihood (i.e. maximised over all parameters except A) is within a particular threshold of the maximum log likelihood over all values of A. The threshold value is calculated as

```
0.5*qchisq(0.95, 1)=1.9207...
```

in the R profile likelihood scripts. The range of A-values that need to be explored is determined by which out of the best fitting Generalised VBGF ($A < 1$), Gompertz ($A = 1$), and supra-exponential ($A > 1$) models are within this threshold value of the best fitting model overall. When the best fitting model is Gompertz or Exponential the confidence interval contains the value A=1, and when Pure Isomorphy has highest (most negative) negative log likelihood (NLL) the confidence interval contains $\frac{2}{3}$. Here are some illustrative examples:

For *Euphasia pacifica*, from Table S1 the best fitting Gompertz ($NLL = -9.57$) and supra-exponential ($NLL = 4.84$) models are all more than 1.9207 away from the best fitting model overall ($A < 1, NLL = -13.32$). The entire confidence interval therefore lies in the range $A < 1$, and both confidence limits can be found using the code in the script “Profile_likelihood_confidence_intervals_Alt1model.R”

For *Daphnia magna,* the negative log likelihood of the best-fitting $A < 1$ model ($NLL = -10.934$) is within 1.9207 of the best fitting model overall (Gompertz, $NLL = -10.934$) so the lower confidence interval is found using the R script "Profile_likelihood_confidence_intervals_Alt1model.R". However, the best fitting $A > 1$ model has a NLL value (-8.093) that is more than 1.9207 above the best fitting model, so the upper confidence limit is A=1.

To run the profile likelihood code, the parameter estimates for the given model obtained from step **(ii)** must be loaded. The rest of the code can then run to obtain upper and lower confidence intervals.

Supplementary Table S1. The negative log likelihood values for the five von Bertalanffy growth function parameterisations: Exponential, Gompertz, Generalised-VBGF, Pure Isomorphy and Supra-exponential for twelve pelagic and benthic invertebrate species. The 'most negative' negative log likelihood values are shown in red for each species and were chosen as the best fitting model.

**Species**	**Negative log likelihood**				
	**Exponential**	**Gompertz**	**Generalised-VBGF**	**Pure Isomorphy**	**Supra-exponential**
Daphnia magna	-8.0929	-10.934	-10.934	-9.996	-8.0929
Euphausia pacifica	4.84441	-9.5694	-13.332	-11.294	4.84441
Pelagia noctiluca	40.3285	6.45159	-1.3867	12.2505	40.3285
Oikopleura dioica	-3.2448	-3.5712	-3.2936	4.48466	-8.0544
Crassostrea gigas	-5.580309	-10.23031	-10.18726	-3.749798	-5.580358
Echinogammarus marinus	22.79021	2.503368	1.056863	1.112388	22.79021
Cherax quadricarinatus	-10.39	-12.284	-12.412	-7.0357	-10.39
Petrarctus demani	2.65892	-8.0612	-10.279	-1.9552	2.16543
Aurelia aurita	-18.807	-18.807	-18.808	-12.789	-27.88
Cyanea capillata	-7.6203	-11.253	-11.303	2.77648	-7.6968
Mytilus edulis	-0.7003123	-3.018598	-3.326951	1.972576	-0.7002971
Sepia officinalis	12.4599	2.66006	2.66006	13.0807	12.4599
